# Internet Survey of Participant Demographics and Risk Factors for Injury in Flyball Dogs

**DOI:** 10.3389/fvets.2019.00391

**Published:** 2019-11-14

**Authors:** Christina Montalbano, Lauri-Jo Gamble, Katherine Walden, Jennifer Rouse, Sabine Mann, Danny Sack, Lauren G. Wakshlag, Justin W. Shmalberg, Joseph J. Wakshlag

**Affiliations:** ^1^Department of Comparative, Diagnostic, and Population Medicine, University of Florida College of Veterinary Medicine, Gainesville, FL, United States; ^2^Department of Clinical Sciences, Cornell University College of Veterinary Medicine, Ithaca, NY, United States; ^3^Homestead Veterinary Services, Haverton, PA, United States; ^4^Department of Population Medicine, Cornell University College of Veterinary Medicine, Ithaca, NY, United States

**Keywords:** canine, flyball, injury, carpus, prevention

## Abstract

**Background:** Injury risk in canine sprinting sports, such as greyhound racing and agility, have been previously documented through various surveys. Flyball, another sprinting canine sport with similar athletic requirements to agility, has yet to be assessed for factors associated with injury. The aim of this study was to determine injury prevalence and assess for risk factors for injury in flyball dogs.

**Methods:** Survey data from 375 flyball participants was collected and analyzed. Data collected included patient-specific variables, equipment use, training/competition practices, and injury occurrence and localization. Univariate analysis was utilized for all variables of interest, followed by backwards nominal logistic regression to identify variables associated with increased risk of injury, with a *p* < 0.05 defined as significant.

**Results:** Thirty-nine percent of dogs incurred at least one injury with 172 injuries reported. Injuries to the limbs were common (30.8% affecting forelimbs, 25.6% affecting hindlimbs), with the paw or nail the most frequently reported injured area (19.2%). Only protective wrap use and age were significantly associated with injury in the final nominal regression model (*p* < 0.01). A biphasic injury rate with more injuries in younger dogs was observed, and injuries peaked by 6 years of age. Use of carpal wraps was positively associated with increased injury risk.

**Conclusions:** These findings suggest an association between younger dogs and greater risk of injury, as well as identify a need to further investigate the utilization of wraps and potential association between injury risk among flyball participants.

## Introduction

Flyball is a growing canine relay sport in which two teams of dogs and handlers compete side-by-side. Teams consist of four dogs, and dogs must traverse four hurdles spaced 3 m apart, retrieve a ball from a spring-loaded flyball box, and return over the hurdles in relay fashion, with the winning team having a lower overall elapsed time for the activity (1, 2). All breeds and mixed breeds are eligible for competing in the sport, with the shortest dog (e.g., height at withers) on the team determining the jump height for the remaining team members. The North American Flyball Association, Inc. reported more than 6,500 registered competing flyball dogs, and over 300 tournaments are held across the continent annually (1). Despite growing interest in the sport, there is a paucity of data regarding participant demographics (both canine and handler), equipment use, training and competition practices, and relative safety of participation.

Risk factors and injury occurrence has been previously evaluated for dogs participating in agility, with a prevalence of ~32% of dogs incurring at least 1 injury with age, breed, sex, level of training and competition, and various other factors associated with increased risk (3–7). The high prevalence of agility injuries in the foot and digits has led to the identification of risk factors suggesting that nail care is essential to prevent digital injuries (8). Injuries associated with racing greyhounds appear to be related to the environment rather than the individual participant, with race speed, distance, and track design associated with variable injury rates (9). Injuries may occur with a lower frequency in canine endurance sports. Recently, injury prevalence has been reported for canicross racing which was lower than agility at ~22%, with breed, running with another dog, and concurrent participation in agility identified as risk factors (10). Although not a prevalence estimate, the orthopedic injury rate of sled dogs competing in a single event of marathon racing was ~15% (11). The athletic requirements of flyball are comparable to agility allowing for similar comparisons regarding injury risk factors (with injury defined as an event where veterinary intervention was considered) and prevalence, and to our knowledge has not previously been reported.

The objectives of this study were (1) to provide descriptive data and statistics on canine participants and injuries incurred in flyball dogs and (2) to identify risk factors associated with patient-specific variables, equipment use, training practices, and competition factors associated with injury. We hypothesized that certain factors are associated with injury including age, sex, breed, spay/neuter status, size of dog, and conditioning and competition practices in flyball dogs.

## Materials and Methods

The web-based survey consisted of 121 questions adapted from a previous agility questionnaire (6) with added questions specifically tailored to flyball participants regarding gear (i.e., harnesses and carpal wraps) and activities (i.e., turn styles etc.). A pilot group of 10 flyball participants were used to validate for accuracy and cognitive interviewing to identify any poorly constructed questions. Revisions of the survey were not deemed necessary, and the survey was distributed via an online survey service (SurveyMonkey Inc., San Mateo, CA, USA. www.surveymonkey.com).

Data collected included dog age (years), sex, age of neuter (<1 year, >1 year, unknown, intact), years in competition, training and conditioning days per week, hours of training per week, use of wraps, harness/collar use, ball-box turning styles, jump height in inches (categorized as 1–5+), dew claw removal, intact/docked tail, size of dog (over or under 12 kg), breed (Border Collie, Border Collie mix, other), and occurrence and localization of injury. Injuries may have occurred at any time in the past, but were restricted to those injuries incurred while participating in competition or training for flyball. Respondents were questioned regarding whether veterinary attention was sought following injury and whether the dog was given time off of sporting activities for recovery. Area injured was reported by respondents with definitive veterinary diagnosis reported when known. Injuries where then categorized based on anatomic location: forelimb and hindlimb digits, carpus, elbow, shoulder, hip, groin (iliopsoas/psoas), stifle, tarsus/hock, back, neck, or undefined/multiple areas.

The survey was completed by respondents in front of a trained veterinary interviewer (LJG, KW) at one of eight flyball events describing the nature of the survey, or respondents could participate online at their convenience after the interviewers supplied them written information on how to complete the survey including relative time taken to complete the survey and use of the data for research purposes. The majority (over half) of the respondents were from the larger national championships in Indianapolis IN, USA. Respondents were asked to fill out only one survey corresponding to a single dog to provide a better cross section of the population on-site, however respondents did have access to the survey after the event in case respondents wanted to complete the survey at home. After being informed their participation was voluntary, completion of the survey was deemed as consent and the survey instrument was exempted by the Cornell University Institutional Review Board due to a lack of human medical or demographic related data in the survey. Responses were collected between March 11, 2017 and December 14, 2017.

### Statistical Analysis

All data was collected from the survey and entered into a Microsoft Excel spreadsheet and double checked for accuracy. A statistical software package was utilized to provide distribution statistics, univariate and logistical regression (JMP Prp 14, SAS Institute, Cary, NC, USA). Distribution of injuries is presented as a percentage of the total population with specific sites of injuries represented as a percent of the injured population. Univariable analysis of injury vs. no injury was determined for all variables and variables with a *p* < 0.2 were included in the initial multivariable nominal logistic regression model with backwards elimination of variables with *p* > 0.1 to achieve the final model. The association between wrap use and anatomic location within the forelimb was assessed by multiple Fisher's Exact analysis across groups with a *p*-value set at 0.05 for significance between injury of the carpus and other forelimb locations (shoulder, elbow, digits).

## Results

After initial assessment of 413 surveys, a total of 375 responses were included in the analysis based on complete information for all of the pertinent questions related to injury. Two hundred and forty (64.0%) surveys were completed in the presence of an investigator. The Border Collie was the most common purebred dog (*n* = 104), followed by the Australian Shepherd (*n* = 25), Whippet (*n* = 18), Labrador (*n* = 16), and Jack Russell Terrier (*n* = 11), with a variety of other purebreds reported in fewer numbers (<10 individuals each). Among mixed breeds (*n* = 129), Border Collie mixes represented almost half of the mixed breed population (*n* = 63), and was thus used as a categorical variable for breed.

Thirty nine percent (*n* = 147) of dogs sustained at least one injury, with 6.7% of dogs (*n* = 25) incurring two or more injuries and 172 total injuries reported. Veterinary attention was sought for diagnosis and treatment in 116 of the 172 injuries (67.4%). Regarding injury localization, 30.8% (*n* = 53) of injuries affected the forelimb, 25.6% (*n* = 44) affected the hindlimb, 14.5% (*n* = 25) affected the back or neck, 9.9% (*n* = 17) affected multiple areas simultaneously, and 19.2% (*n* = 33) were unspecified/non-specific (e.g., “leg”). Injuries were further localized to the fore paw pad, nail, or digit most frequently (*n* = 33); followed by shoulder (*n* = 26), neck, back, or tail (*n* = 23), iliopsoas/groin musculature (*n* = 17), carpus (*n* = 6) and elbow (*n* = 3).

Univariable analysis of the injury vs. no-injury data provided four variables with a regression *p* < 0.2 including: wraps use, age, spay/neuter age, and years of competition (Tables 1–3). Final multivariable nominal logistic regression showed only two remaining variables in the model being age of dog [*p* < 0.001 (OR 0.78–95% CI 0.67–0.93); Table 4] and use of wraps [*p* = 0.002 (OR 2.46–95% CI 1.52–3.94); Table 4]. Younger dogs, specifically those <6 years of age, were found to be at greater risk for injury. There was a positive association between the use of wraps and increased injury risk. Individual Fisher's exact tests of carpal injury vs. elbow, shoulder, and digit were performed (Table 5) and there were no differences found between these anatomic locations (*p* > 0.18).

**Table 1:** Univariate analysis assessing canine subject demographic/anatomic factors vs. injury status.

**Factor**	**Uninjured**	**Injured**	***P*-value**
Number of dogs (%)	228 (60.8)	147 (39.2)	
Sex (No. [%])			0.25
M	30 (13.2)	14 (9.5)	
MN	85 (37.3)	66 (44.9)	
F	22 (9.6)	9 (6.1)	
FS	91 (39.9)	58 (39.5)	
Age (Mean + SD)	6.2 ± 2.7	4.7 ± 2.8	<0.01
Age at neuter (No. [%])			0.16
<1	79 (34.6)	61 (41.5)	
>1	91 (39.9)	56 (38.1)	
Intact	52 (22.8)	23 (15.6)	
Unknown	6 (2.6)	7 (4.8)	
Breed (No. [%])			0.51
Border Collie	54 (23.7)	44 (29.9)	
BC Mix	35 (15.4)	24 (16.3)	
Other purebred or mix	139 (61.0)	79 (53.7)	
Dog size (No. [%])			0.31
<12 kg	61 (26.8)	33 (22.4)	
>12 kg	167 (73.2)	113 (76.9)	
Docked tail (No. [%])			0.26
Yes	40 (17.5)	18 (12.2)	
No	188 (82.5)	129 (87.9)	
Dew claws (No. [%])			0.79
Intact	168 (73.7)	111 (75.5)	
Removed	50 (26.3)	36 (24.5)	

**Table 2:** Univariate analysis assessing subject running style and specialized equipment use vs. injury status.

**Factor**	**Uninjured**	**Injured**	***P*-value**
Number of dogs (%)	228 (60.8)	147 (39.2)	
Jump height 1–5+			0.43
1	70 (30.7)	41 (27.9)	
2	46 (20.2)	25 (17.0)	
3	64 (28.1)	54 (36.7)	
4	31 (13.5)	20 (13.5)	
5+	17 (7.5)	7 (4.8)	
Turn direction (No. [%])			0.59
Clockwise	124 (54.4)	74 (50.3)	
Counterclockwise	104 (45.6)	73 (49.7)	
Turn style (No. [%])			0.51
2 foot	10 (4.4)	10 (6.8)	
3 foot	28 (12.3)	20 (13.6)	
4 foot	185 (81.1)	111 (75.5)	
Mix	5 (2.2)	6 (4.1)	
Use of wraps (No. [%])			<0.01
Yes	80 (35.1)	72 (49.0)	
No	148 (64.9)	75 (51.0)	
Equipment (No. [%])			0.34
None	52 (22.8)	41 (27.9)	
Harness	62 (27.2)	50 (34.0)	
Flat collar	65 (28.5)	34 (23.1)	
Martingale	45 (19.7)	21 (14.3)	

**Table 3:** Univariate analysis assessing practice and competition frequency vs. injury status.

**Factor**	**Uninjured**	**Injured**	***P*-value**
Number of dogs (%)	228 (60.8)	147 (39.2)	
Years competing (Mean ± SD)	3.9 ± 2.8	5.2 ± 2.7	<0.01
Trials/year (No. [%])			0.26
1	36 (15.8)	11 (7.5)	
2	114 (50.0)	67 (45.5)	
3	57 (25.0)	51 (34.7)	
4+	21 (9.2)	18 (12.3)	
Practice sessions/weak (No. [%])			0.83
1	152 (66.7)	99 (67.3)	
2	21 (9.2)	18 (12.2)	
3	4 (1.7)	1 (0.6)	
4+	51 (22.4)	29 (19.7)	
Non-flyball conditioning house/weak (No. [%])			0.21
1	51 (22.4)	27 (18.4)	
2	43 (18.9)	41 (27.9)	
3	130 (57.0)	77 (52.3)	
4+	4 (1.7)	2 (1.3)	
Flyball practice hours/weak (No. [%])			0.24
1	100 (43.9)	65 (44.2)	
2	59 (25.9)	34 (14.9)	
3	37 (16.2)	29 (19.7)	
4+	32 (14.0)	19 (12.9)	

**Table 4:** Results of final multivariable nominal regression model showing the effects of the remaining variables associated with injury in flyball dogs (*n* = 375).

**Variable**	**β (SE)**	**aOR**	**Confidence interval**	***P*-value**
			**(5th−95th)**	
Current Age (yrs)	−0.23 (0.08)	0.78	(0.67–0.93)	<0.01
Wraps	0.45 (0.12)			<0.01
Yes		2.46	(1.52–3.94)	
No		-Referent-		

*aOR, adjusted odds ratio*.

**Table 5:** Forelimb injury categorization (digit, carpus, elbow shoulder) and Fisher's Exact Test–no *p*-value was below *p* < 0.18.

	**Digits**	**Carpus**	**Elbow**	**Shoulder**
Wrap	22	2	1	15
No wraps	11	4	2	11

## Discussion

The prevalence of injury in flyball dogs (39%) appears to be similar to that reported in agility dogs (32–33%) in owner dependent survey responses (3–8). This may be due to the similarities between the two sports. Surprisingly, there were very few factors associated with increased risk of injury when compared to factors associated with injury in agility dogs, with only younger age and the use of wraps reaching significance. In agility, major risk factors across studies appear to be breed related (Border Collie) and age associated (4, 5, 7, 8). Other risk factors such as having a full tail and potentially early neutering/spaying having associations do not appear in this flyball cohort, which may be due to flyball requiring fewer specialized tasks as opposed to agility (6) which requires many different tasks. Both agility and flyball require dogs to jump hurdles at top speed with tight turns. However, flyball requires just the one turn with a straight course of hurdles and agility incorporates many turns as well as a variety of other equipment contact which is thought to contribute directly to agility injury (3, 5). While an association between injury and contact with hurdles has been identified in agility dogs, we did not specifically look for contact with the flyball box or a teammate collision as a specific risk factor, which warrants further investigation as to whether contact with obstacles or hurdles in both sports leads to the similar injuries.

The hurdle height determinant in flyball is unique when compared to agility. In agility, jump height is determined by the competing dog's height at the withers, with dogs competing in a class of similar-height dogs. In contrast, flyball sets the hurdle height of a team based on the shortest dog competing, measured either by the height at the withers or length from point of the elbow to the accessory carpal bone (1, 2). This could theoretically place smaller dogs at greater risk of injury, as a previous study in agility activities found increasing jump height to be associated with greater scapulohumeral joint flexion and proposed stretching of the biceps brachii and supraspinatus musculature (12). Distance between hurdles may also alter jumping and landing kinematics (13); while jump distance is set at 3 m in flyball, the relative distance vs. dog size varies considerably. Our study did not find an association between relative dog size (<12 vs. >12 kg), jump height, and injury; thus, it is likely that the described changes in joint angles are clinically unimportant during flyball activity. Another study found significant increases to ground reaction forces of up to 4.5 times body weight of the landing forelimbs when a dog lands a jump at high speeds (14). Based on these prior findings one could suppose that faster average flyball course times would be associated with injury risk which was not evident in our study.

The prevalence of injury may be slightly higher among flyball dogs when compared to sled dogs and canicross (10, 11). These sports are both endurance races, with dogs maintaining slower speeds for long distances, compared to the sprint of flyball competitors. Sled dog racing and canicross are also performed across relatively straight paths with no sharp turns, while flyball requires a quick 180° turnabout upon retrieval of the ball from the box. These differences between the sports may explain the difference in injury rates. It must also be mentioned that the sled dog study assessed injury rate during an individual race as an actual incidence report and not as prevalence reported over a population of dogs of varying ages, making comparisons difficult.

Similar to injury localizations reported for agility, greyhound racing, and canicross, there appears to be a higher prevalence of forelimb injury than hindlimb injury in this flyball cohort (5, 7, 9, 10). This may be a reflection of the average forelimb vs. hindlimb weight distribution. Injuries to the paw pad/foot or shoulder were most common among this flyball population, with surprisingly few carpal injuries, despite the perception of owners and sporting enthusiasts that hyperextension of carpi on impact with the flyball platform is problematic. Injury occurrence to the paw pad or foot region in this study (19.2% of all injuries) was similar to that of a previous study looking specifically at digital injuries in agility dogs (23.7%) (8). Risk factors included nail length, absence of front dewclaws, and greater weight to height ratio among affected agility dogs; further study is needed to understand foot/digit injuries in flyball dogs to identify specific risk factors and potential methods for prevention.

Experience in a sport is often perceived to affect injury risk and occurrence rates, although measurement of experience is not precise. This study assessed age, number of years training, and time dedicated to flyball training, with only age being significantly associated with injury. Studies in other sporting dogs have found similar associations when looking at either age or years of experience specific to that sport (4, 7, 8, 10). It is unclear whether the association between increasing age and decreasing risk of injury found in this study is an indirect indicator of greater experience in the sport or other factors, but it is apparent that injuries were more commonly reported early in a dogs career, with risk decreasing in older dogs (>6 years). One possible explanation is that dogs with chronic injuries may not be represented due to discontinuation of the sport with only sound older dogs attending the competitions from which our survey solicited. Along the same tenets, increased conditioning and hours of athletic activity or competition may be indirect markers of sporting experience and associated with an altered risk of injury, although no association was found in the present study. Alternatively, if participation in the sport itself belies the injury occurrence, greater time spent engaged in activity would be expected to be associated with increased injury risk; again, no such association was identified.

The use of wraps while training or competing was identified to be common in flyball dogs. Several types of wraps were utilized, from supportive tape wraps (most common) used in attempts to prevent carpal hyperextension, or more extensive commercially produced protective wraps to prevent carpal injury and paw pad abrasions or to improve traction. The association between use of wraps and increased injury risk is concerning; however, the survey could not be used to determine causality because the question did not ask whether wraps were used for previous injury or as a preventive measure. In human athletics it is relatively well-known that ankle wrapping is associated with fewer tarsal injuries and is advocated for chronic injury, while it is also described that kinematics of other leg joints (knee and hip) are altered leading to theories that ankle wrapping may lead to other lower extremity injuries (15–17). This warrants future investigation into the use of carpal wraps and their relationship to injury in the carpus as well as the proximal joints in competitive flyball dogs.

The lack of significance in risk of injury among Border Collies was in contrast to several agility studies identifying increased injury risk in the breed with odds ratios ranging from 1.7 to 10.5 (4, 5, 8). Border Collies were the most frequently reported purebred participants as identified in this flyball cohort and the previous agility surveys (3–8). It is unknown why the breed is at greater risk of injury in agility, but has been proposed that high drive and ability to rapidly alter speed and direction are problematic (4). Exactly why the breed appears more adept at performing flyball without injury is unclear, yet as previously discussed the actions required in flyball are far more limiting and repetitive than in agility.

This study is subject to a number of limitations. Recall bias is expected as the respondent provides information without independent verification of the responses. Selection bias could occur if handlers of flyball dogs who incurred injury were more likely to complete the survey due to greater personal interest in study results. The study was limited to flyball competitors at specific events, thus limiting respondents to only competitive dogs across differing ages and only healthy dogs that are attending events. In cases of injury, definitive veterinary diagnosis was not required, and information regarding necessary care, required time off, and ability and success upon return to sport was not within the scope of this investigation. However, 2/3rds of injuries were reportedly presented to a veterinarian for diagnosis and appropriate treatment, thus increasing the probability that injury localization was appropriate. In addition, specifics of timing as it relates to competition or training for flyball, or where in the course injury occurred (start, ball box, hurdle, etc.) were insufficient to draw conclusions about these specifics regarding injury.

In conclusion, prevalence rate of injury among flyball dogs was similar to the comparable sport of agility, but possibly higher than endurance type sports including sled dog racing and canicross. Prevalence in forelimb vs. hindlimb injury compared to other sports was no different, with the highest occurrence of injury in the digit or pad, similar to agility. The overall lack of associations between various presumed risk factors suggest the relative safety of the sport, and further investigation regarding the practices and training or competing conditions in flyball dogs as compared to agility dogs is warranted, due to differences in risk factor. In addition our findings surrounding the use of wraps suggests further study is needed on the utility of carpal and paw bracing during this unique sport.

## Data Availability Statement

The datasets generated for this study are available on request to the corresponding author.

## Author Contributions

JW, JR, and L-JG contributed in the genesis of the study idea, survey and study design. CM, KW, LW, L-JG, and DS collected data and performed data entry and analysis. SM, JW, JS, and CM took part in the statistical analysis and drafted the manuscript. All authors contributed to the manuscript preparation and revisions for submission.

### Conflict of Interest

The authors declare that the research was conducted in the absence of any commercial or financial relationships that could be construed as a potential conflict of interest.
