# Supplementary material for: Internet Survey of Participant Demographics and Risk Factors for Injury in Flyball Dogs
Source: Front Vet Sci. 2019 Nov 14;6:391. doi: 10.3389/fvets.2019.00391 (PMC6874169; doi:10.3389/fvets.2019.00391)
Supplement: Supplementary file 1 [file Data_Sheet_1.pdf]

Copy of Canine Flyball Injury and Nutrition

SUMMARY → DESIGN SURVEY → PREVIEW & SCORE → COLLECT RESPONSES → ANALYZE RESULTS → PRESENT RESULTS

NEXT →

QUESTION BANK

Search for questions

- Recommended Questions
- Previously Used Questions
- All Categories
- Community
- Customer Feedback
- Customer Satisfaction
- Demographics
- Education
- Events
- **Healthcare**
- Human Resources
- Industry Specific
- Just for Fun

P1: Dog Inform...

Page Logic | More Actions

Cornell University
College of Veterinary Medicine

Copy of Canine Flyball Injury and Nutrition

Dog Information

1. What is the name of your dog? If you have multiple dogs, chose one specific dog.

2. What is your team name?

3. What breed(s) is your dog?

4. What sex is your dog?

- Intact Male
- Neutered Male
- Intact Female
- Spayed Female

5. How old was your dog when spayed/neutered?

5. How old was your dog when spayed/neutered?

6. How old is your dog (in years)?

7. How much does your dog weigh in pounds?

New version available!

Saving changes...

8. What is your dog's height in inches (at withers/shoulder)?

9. How old was your dog when adopted/purchased?

10. Where did you adopt/purchase your dog?

- From a breeder
- From a family member/friend
- From a shelter or a rescue group
- Other (please specify)

11. At what age did your dog start flyball training (including hurdles and box, not simply general training)?

12. At what age did your dog start flyball competition?

NEW QUESTION

or Copy and paste questions

Next

Powered by
SurveyMonkey
See how easy it is to create a survey.

New version available!

Saving changes...

NEW PAGE

P2: Flyball Co...

Page Logic

More Actions

Cornell University
College of Veterinary Medicine

Copy of Canine Flyball Injury and Nutrition

Flyball Competition

13. Which flyball organizations does your dog compete in? (check all that apply)

- [ ] NAFA
- [ ] U-FLI
- [ ] Other (please specify)

14. (If racing in NAFA, which class of competition does your dog perform in?)

- [ ] Regular
- [ ] Multi-breed
- [ ] Veterans

- [ ] Open
- [ ] Not competing in NAFA
- [ ] Other (please specify)

15. (If racing in U-FLI, which class of competition does your dog perform in?)

New version available!

Saving changes...

- [ ] Standard
- [ ] Variety
- [ ] Singles
- [ ] Pairs
- [ ] Not competing in U-FLI
- [ ] Other (please specify)

16. Has your dog earn any title? If so, what title or points?

17. What jump height is your dog competing at (in inches)?

18. Which way does your dog turn (from the dogs perspective)?

- [ ] Right (clockwise)
- [ ] Left (counter clockwise)

19. Describe your dog's turning pattern

- ( ) 2 pawed
- ( ) 3 pawed
- ( ) 4 pawed
- ( ) Other (please specify)

20. What is your dog's average time (in seconds)?

21. What is your dog's best time (if known)?

22. How many years has you

New version available!

Saving changes...

23. On average, how many trials do you attend annually?

24. On average, how often does your dog run in a tournament?

25. Describe the average percentage (%) of trials/training performed on:

Outdoor grass

Indoor turf

Indoor mats over concrete flooring

Other

26. What does your dog wear while running?

- [ ] Naked
- [ ] Harness
- [ ] Martingale

Other (please specify)

27. Does your dog wear wraps?

- No
- Yes. If so, what type (skid boots, powerflex, etc.)? What do you wrap? Why?

NE

New version available!

Saving changes...

or Copy and paste questions

Prev Next

Powered by
SurveyMonkey
See how easy it is to create a survey.

NEW PAGE

P3: Maintenan... Page Logic More Actions

Cornell University
College of Veterinary Medicine

Copy of Canine Flyball Injury and Nutrition

Maintenance and conditioning program

28. How often does your dog attend flyball practice?

- 3+ times per week
- Twice a week
- Once a week
- Every other week
- Other (please specify)

29. What sorts of drills do yo

New version available!

Saving changes...

- Box work
- Power jumping
- Lineups
- Other (please specify)

30. Do you condition your dog outside of flyball practice? If so how often?

- No
- Yes, 3+ times per week
- Yes, twice per week
- Yes, once per week
- Yes, every other week
- Other (please specify)

31. On average, how many days per week do you train doing each of the following? (total time spent at practice, not only burst of activity)

Flyball

Conditioning/Cro
ss-Training

Obedience

Agility

Strength/Flexibili
ty

Other

32. On average, how many hours per week do you train when doing each of the following activiti[illegible]

New version available!

Saving changes...

Flyball

Conditioning/Cro
ss-Training

Obedience

Agility

Strenght/Flexibili
ty

Other

33. Does your dog compete in any other canine sports? (check all that apply)

- [ ] Dock Dogs
- [ ] Herding
- [ ] Competitive Obedience
- [ ] Rally Obedience
- [ ] Agility
- [ ] Schutzhund
- [ ] Disc Dog
- [ ] Other (please specify)
- [ ] Field Trials
- [ ] Hunt Tests
- [ ] Mushing
- [ ] Dirt Dogs
- [ ] Lure coursing
- [ ] None

34. Does your dog participate in any of the following treatments for a chronic injury or for wellness?

- [ ] Physical rehabilitation
- [ ] Laser therapy
- [ ] Therapeutic ultrasound
- [ ] TENS
- [ ] Electromagnetic field device (PEMF)
- [ ] Acupuncture
- [ ] Chiropractic
- [ ] Massage and manual therapy
- [ ] Heat and/or cold packing
- [ ] Brace, wrap, orthotic or other immobilization device
- [ ] None
- [ ] Other (please specify)

New version available!

Saving changes...

NEW QUESTION

or Copy and paste questions

Prev Next

Powered by
SurveyMonkey
See how easy it is to create a survey.

NEW PAGE

P4: Nutrition

Page Logic More Actions

Cornell University
College of Veterinary Medicine

Copy of Canine Flyball Injury and Nutrition

New version available!

Saving changes...

Nutrition

35. What do you feed your dog primarily (75% or greater as main meals):

- Commercial dog food (primarily kibble, canned)
- Non-traditional Commercial dog food (primarily raw, freeze dried, cooked food like Freshpet)
- Home-prepared raw diet
- Home prepared cooked diet
- A combination of both commercial kibble/canned and cooked home-prepared food
- A combination of both commercial kibble/canned and commercial or home prepared raw

NEW QUESTION

or Copy and paste questions

Prev Next

Powered by
SurveyMonkey
See how easy it is to create a survey.

NEW PAGE

P5: Commercia...

Page Logic

More Actions

Cornell University
Colleg [illegible]

New version available!

Saving changes...

Copy of Canine Flyball Injury and Nutrition

Commercial Dog Food

36. What do you currently feed your dog? (Today)

Brand(s)?

Formula(s)?

37. How much commercial food (cups, cans, pounds) do you feed your dog per day?

38. How long have you been feeding this(these) brand(s)?

39. Do you add any human food/table scraps to your dog's meals (non-prepared meals)?

- Yes
- No

40. (Approximately how much human food/table scraps does your dog receive each day with meals?)

[illegible]

41. (In general, which do you provide the most of?)

- Meat
- Vegetables
- Seafood
- Dairy
- Carbohydrates/Grains
- None given
- Fruits
- Other (please specify)

New version available!

Saving changes...

NEW QUESTION

or Copy and paste questions

Prev Next

Powered by
SurveyMonkey
See how easy it is to create a survey.

NEW PAGE

P6: Home-Prep... Page Logic More Actions

Cornell University
College of Veterinary Medicine

Copy of Canine Flyball Injury and Nutrition

Home-Prepared Food

42. If feeding a home prepared raw or cooked diet as the primary meal where did you get the formulation for preparing this food?

- Internet resource
- Veterinarian
- Veterinary Nutritionist
- Book
- Breeder
- Friend/Colleague
- other

New version available!

Saving changes...

NEW QUESTION

or Copy and paste questions

Prev Next

Powered by
SurveyMonkey
See how easy it is to create a survey.

NEW PAGE

P7: Combinati...

Page Logic

More Actions

Cornell University
College of Veterinary Medicine

Copy of Canine Flyball Injury and Nutrition

Combination Feeding: Commercial Dog Food

43. IF feeding some home prepared raw or cooked food where did you get the formulation for the prepared food?

- Internet Resource
- Veterinarian
- Veterinary Nutirtionist
- Breeder
- Book
- Friend/Colleague
- Other

New version available!

Saving changes...

NEW QUESTION

or Copy and paste questions

Prev Next

Powered by
SurveyMonkey
See how easy it is to create a survey.

NEW PAGE

P8: Combinati...

Page Logic More Actions

Cornell University
College of Veterinary Medicine

Copy of Canine Flyball Injury and Nutrition

Combination Feeding: Home

New version available!

Saving changes...

44. If feeding a home prepared diet how did you get the recipe currently used?

- ◯ Internet resource
- ◯ Veterinarian advice
- ◯ Veterinary Nutritionist
- ◯ Breeder
- ◯ Book
- ◯ Friend/Colleague
- ◯ Other

NEW QUESTION

or Copy and paste questions

Prev Next

Powered by
SurveyMonkey®
See how easy it is to create a survey.

NEW PAGE

P9: General Fe...

Page Logic More Actions

Cornell University
College of Veterinary Medicine

New version available!

Copy of Canine Flyball Inj

Saving changes...

General Feeding

45. How many times do you feed your dog per day?

- 1 meal
- 2 meals
- 3 meals
- >3 meals
- Free-fed
- Other (please specify)

46. Do you feed-restrict on competition days?

- Yes
- No

47. Do you change feeding strategies or foods based on season (winter, spring, summer, fall)?

- Yes
- No

If yes, please explain

48. Do you change feeding strategies or foods based on current training or activity level?

- Yes
- No

If yes, please explain

49. What are the 3 most important factors to you when choosing a dog food?

- Protein content
- Carbohydrate content
- Fat content
- Fiber content
- No byproducts
- Fruit/Vegetable content
- Organic
- Most important factors 1-3

New version available!

Saving changes...

- Corn, Soy, Wheat-free
- Price
- Brand
- Word of Mouth/Recommendation
- I don't know

NEW QUESTION

or Copy and paste questions

Prev Next

Powered by
SurveyMonkey
See how easy it is to create a survey.

NEW PAGE

P10: Treats

Page Logic More Actions

Cornell University
College of Veterinary Medicine

Copy of Canine Flyball Injury and Nutrition

New version available!

Treats

Saving changes...

50. What are your top 3 treat choices?

1

2

3

51. Approximately what amount of each treat do you give your dog per day, including training treats?

NEW QUESTION

or Copy and paste questions

Prev Next

Powered by
SurveyMonkey
See how easy it is to create a survey.

NEW PAGE

P11: Suppleme...

Page Logic More Actions

Cornell University
Colleg

New version available!

Saving changes...

Copy of Canine Flyball Injury and Nutrition

Supplements

52. Do you give your dog any (if not giving, please leave blank):

Joint supplements (eg glucosamine/chondroitin, fish oil)

Focus supplements (eg ginko baloba, senilife)

Skin/coat supplements (eg fatty acid, vitamins)

Performance supplements (eg creatine, maltodextrin, glucose repletion)

Heat stress supplements (eg glycerol, electrolytes)

Stress diarrhea supplements (eg fibers, slippery elm, metronidazole)

Antioxidant supplements (eg Vitamin E, Vitamin C)

New version available!

Saving changes...

Vitamin supplements (eg multivitamin, Pettabs)

Anti-inflammatory drugs (eg Carprofen/Meloxicam/Previcox/Deramaxx, Aspirin)

Other

53. Does your dog get any supplements during competitions to enhance performance? (check all that apply)

- [ ] Electrolytes
- [ ] Glucose supplement
- [ ] Fat supplement
- [ ] Antioxidants
- [ ] Protein supplement
- [ ] None
- [ ] Other (please specify)

NEW QUESTION

or Copy and paste questions

Prev Next

New version available!

Saving changes...

See how easy it is to create a survey.

NEW PAGE

P12: Injury Hist...

Page Logic More Actions

Cornell University
College of Veterinary Medicine

Copy of Canine Flyball Injury and Nutrition

Injury History

54. Is your dog's tail docked?

- Yes
- No

55. Is your dog naturally bob-tailed or was it docked?

- Natural bob-tail
- Docked
- Full length

56. Does your dog have front dew-claws?

[illegible]

- Yes
- No

57. Has your dog ever been injured while participating in or training for flyball?

- Yes
- No
- I don't know

New version available!

Saving changes...

NEW QUESTION

or Copy and paste questions

Prev Next

Powered by
SurveyMonkey
See how easy it is to create a survey.

NEW PAGE

P13: Injury Hist... Page Logic More Actions

Cornell University
College of Veterinary Medicine

Copy of Canine Flyball Injury and Nutrition

Injury History 1

58. After you recognized the injury, what course did you pursue?

- [ ] Continued activity as usual
- [ ] Enforced time off
- [ ] Sought veterinary attention
- [ ] Other (please specify)

New version available!

Saving changes...

59. What body part did your dog injure (i.e wrist, knee, elbow, shoulder, hock, hip back)?

60. What type of injury occurred?

- ( ) Muscle strain/pull/tear
- ( ) Tendon strain/pull/rupture
- ( ) Ligament sprain/rupture
- ( ) Fracture
- ( ) Dislocation
- ( ) I don't know
- ( ) Other (please specify)

61. What was the diagnosis, if known?

62. How many times has this injury occurred?

63. Please categorize the injury.

- ( ) Acute and immediately treatable (like fracture)
- ( ) Chronic (like arthritis)
- ( ) I don't know

64. What treatment/repairs were performed?

65. Did treatment include home exercises and/or formal physical rehabilitation (underwater treadmill therapy, laser therapy, acupuncture, etc.)?

- Yes, both
- Yes, only at home exercises
- Yes, only formal physical rehal
- No
- I don't know

New version available!

Saving changes...

66. If formal physical rehabilitation was recommended, which modalities were performed

- Underwater treadmill therapy
- Land exercises
- Laser therapy
- Therapeutic ultrasound
- Shockwave therapy
- TENS
- Acupuncture
- Chiropractic
- Massage and manual therapy
- Heat and/or cold
- Regenerative medicine (stem cells, PRP)
- Other joints injections (corticosteroids, HA, etc.)
- Brace, wrap, orthotic or other immobilization device
- Other (please specify)

67. Is there anything else you would like to let us know about this injury?

68. Has your dog sustained another injury?

- Yes
- No

NEW QUESTION

or Copy

New version available!

Saving changes...

Prev Next

Powered by
SurveyMonkey
See how easy it is to create a survey.

NEW PAGE

P14: Injury Hist...

Page Logic More Actions

Cornell University
College of Veterinary Medicine

Copy of Canine Flyball Injury and Nutrition

Injury History 2

69. After you recognized the injury, what course did you pursue?

- [ ] Continued activity as usual
- [ ] Enforced time off

- [ ] Sought veterinary attention
- [ ] Other (please specify)

70. What body part did your dog injure?

71. What type of injury occu

New version available!

Saving changes...

- ( ) Muscle strain/pull/tear
- ( ) Tendon/ strain/pull/rupture
- ( ) Ligament sprain/rupture
- ( ) Fracture
- ( ) Dislocation
- ( ) I don't know
- ( ) Other (please specify)

72. What was the diagnosis, if known?

73. How many times has this injury occurred?

74. Please categorize the injury.

- ( ) Acute, and immediately treatable (like fracture)
- ( ) Chronic (like arthritis)
- ( ) I don't know

75. What treatment/repairs were performed?

76. Did treatment include home exercises and/or formal physical rehabilitation (underwater treadmill therapy, laser therapy, acupuncture, etc.)?

- ◯ Yes, both
- ◯ Yes, only at home exercises
- ◯ Yes, only formal physical rehabilitation
- ◯ No
- ◯ I don't know

77. If formal physical rehabil[illegible] modalities were performed

New version available!

Saving changes...

- ☐ Underwater treadmill therapy
- ☐ Land exercises
- ☐ Laser therapy
- ☐ Therapeutic ultrasound
- ☐ Shockwave therapy
- ☐ TENS
- ☐ Acupuncture
- ☐ Chiropractic
- ☐ Massage and manual therapy
- ☐ Heat and/or cold
- ☐ Regenerative medicine (stem cells, PRP)
- ☐ Other joints injections (corticosteroids, HA, etc.)
- ☐ Brace, wrap, orthotic or other immobilization device
- ☐ Other (please specify)

78. Is there anything else you would like to let us know about this injury?

79. Has your dog sustained another injury?

- ◯ Yes
- ◯ No

NEW QUESTION

or Copy and paste questions

Prev Next

New version available!

Saving changes...

Powered by
SurveyMonkey
See how easy it is to create a survey.

NEW PAGE

P15: Injury Hist... Page Logic More Actions

Cornell University
College of Veterinary Medicine

Copy of Canine Flyball Injury and Nutrition

Injury History 3

80. After you recognized the injury, what course did you pursue?

- [ ] Continued activity as usual
- [ ] Enforced time off
- [ ] Sought veterinary attention
- [ ] Other (please specify)

81. What body part did your dog injure?

82. What type of injury occurred?

- Muscle strain/pull/tear
- Tendon/ strain/pull/rupture
- Ligament sprain/rupture
- Fracture
- Dislocation
- I don't know
- Other (please specify)

New version available!

Saving changes...

83. What was the diagnosis, if known?

84. How many times has this injury occurred?

85. Please categorize the injury.

- Acute, and immediately treatable (like fracture)
- Chronic (like arthritis)
- I don't know

86. What treatment/repairs were performed?

87. Did treatment include home exercises and/or formal physical rehabilitation (underwater treadmill therapy, laser therapy, acupuncture, etc.)?

- Yes, both
- Yes, only at home exercises
- Yes, only formal physical rehabilitation
- No

- ◯ I don't know

88. If formal physical rehabilitation was recommended, which modalities were performed

- ☐ Underwater treadmill therapy
- ☐ Land exercises
- ☐ Laser therapy
- ☐ Therapeutic ultrasound
- ☐ Shockwave therapy
- ☐ TENS
- ☐ Acupuncture
- ☐ Chiropractic
- ☐ Massage and manual therapy
- ☐ Heat and/or cold
- ☐ Regenerative medicine (stem cells, PRP)
- ☐ Other joints injections (corticosteroids, HA, etc.)
- ☐ Brace, wrap, orthotic or other immobilization device
- ☐ Other (please specify)

New version available!

Saving changes...

89. Is there anything else you would like to let us know about this injury?

90. Has your dog sustained another injury?

- ◯ Yes
- ◯ No

NEW QUESTION

or Copy and paste questions

Prev Next

Powered by
SurveyMonkey
See how easy it is to create a survey.

New version available!

Saving changes...

NEW PAGE

P16: Injury Hist... Page Logic More Actions

Cornell University
College of Veterinary Medicine

Copy of Canine Flyball Injury and Nutrition

Injury History 4

91. After you recognized the injury, what course did you pursue?

- [ ] Continued activity as usual
- [ ] Enforced time off
- [ ] Sought veterinary attention
- [ ] Other (please specify)

92. What body part did your dog injure?

93. What type of injury occurred?

- ( ) Muscle strain/pull/tear

- Tendon/ strain/pull/rupture
- Ligament sprain/rupture
- Fracture
- Dislocation
- I don't know
- Other (please specify)

New version available!

Saving changes...

94. What was the diagnosis, [illegible]

95. How many times has this injury occurred?

96. Please categorize the injury.

- Acute, immediately treatable (like fracture)
- Chronic (like arthritis)
- I don't know

97. What treatment/repairs were performed?

98. Did treatment include home exercises and/or formal physical rehabilitation (underwater treadmill therapy, laser therapy, acupuncture, etc.)?

- Yes, both
- Yes, only at home exercises
- Yes, only formal physical rehabilitation
- No
- I don't know

99. If formal physical rehabilitation was recommended, which modalities were performed

- Underwater treadmill therapy

- [ ] Land exercises
- [ ] Laser therapy
- [ ] Therapeutic ultrasound
- [ ] Shockwave therapy
- [ ] TENS
- [ ] Acupuncture
- [ ] Chiropractic
- [ ] Massage and manual therapy
- [ ] Heat and/or cold
- [ ] Regenerative medicine (stem cells, PRP)
- [ ] Other joints injections (corticosteroids, HA, etc.)
- [ ] Brace, wrap, orthotic or other immobilization device
- [ ] Other (please specify)

New version available!

Saving changes...

100. Is there anything else you would like to let us know about this injury?

101. Has your dog sustained another injury?

- ( ) Yes
- ( ) No

NEW QUESTION

or Copy and paste questions

Prev Next

Powered by

SurveyMonkey
See how easy it is to create a survey.

NEW PAGE

P17: Injury Hist...

New version available!

Saving changes...

Cornell University
College of Veterinary Medicine

Copy of Canine Flyball Injury and Nutrition

Injury History 5

102. After you recognized the injury, what course did you pursue?

- [ ] Continued activity as usual
- [ ] Enforced time off
- [ ] Sought veterinary attention
- [ ] Other (please specify)

103. What body part did your dog injure?

104. What type of injury occurred?

- ( ) Muscle strain/pull/tear
- ( ) Tendon/ strain/pull/rupture
- ( ) Ligament sprain/rupture
- ( ) Fracture
- ( ) Dislocation
- ( ) I don't know

- ◯ Other (please specify)

105. What was the diagnosis, if known?

106. How many times has thi

New version available!

Saving changes...

107. Please categorize the injury.

- ◯ Acute, and immediately treatable (like fracture)
- ◯ Chronic (like arthritis)
- ◯ I don't know

108. What treatment/repairs were performed?

109. Did treatment include home exercises and/or formal physical rehabilitation (underwater treadmill therapy, laser therapy, acupuncture, etc.)?

- ◯ Yes, both
- ◯ Yes, only at home exercises
- ◯ Yes, only formal physical rehabilitation
- ◯ No
- ◯ I don't know

110. If formal physical rehabilitation was recommended, which modalities were performed

- ☐ Underwater treadmill therapy
- ☐ Land exercises
- ☐ Laser therapy
- ☐ Therapeutic ultrasound
- ☐ Shockwave therapy
- ☐ TENS

- Acupuncture
- Chiropractic
- Massage and manual therapy
- Heat and/or cold
- Regenerative medicine (stem cells, PRP)
- Other joints injections (corticosteroids, HA, etc.)
- Brace, wrap, orthotic or other i[illegible]
- Other (please specify)

New version available!

Saving changes...

111. Is there anything else you would like to let us know about this injury?

112. Has your dog sustained another injury?

- Yes
- No

NEW QUESTION

or Copy and paste questions

Prev Next

Powered by
SurveyMonkey
See how easy it is to create a survey.

NEW PAGE

P18: More than...

Page Logic | More Actions

Cornell University
College of Veterinary Medicine

New version available!

Copy of Canine Flyball Inj

Saving changes...

More than 5 Injuries

113. Please explain.

NEW QUESTION

or Copy and paste questions

Prev | Next

Powered by
SurveyMonkey
See how easy it is to create a survey.

NEW PAGE

P19: Responde...

Page Logic | More Actions

Cornell University
College of Veterinary Medicine

Copy of Canine Flyball Injury and Nutrition

Respondent Information (thi

New version available!

Saving changes...

114. Gender

- Male
- Female
- Prefer not to answer

115. Your Age Range

116. Socioeconomic Level

117. What is your home state?

118. Do you consider yourself to be an athlete?

- Yes
- No
- Prefer not to answer

NEW QUESTION

or Copy and paste questions

Prev Next

Powered by
SurveyMonkey
See how easy it is to create a survey.

New version available!

Saving changes...

NEW PAGE

P20: Weblink v... Page Logic More Actions

Cornell University
College of Veterinary Medicine

Copy of Canine Flyball Injury and Nutrition

Weblink vs on site

119. Are you filling this survey in the presence of the interviewer from Cornell or own your own?

- With the interviewer
- On my own

NEW QUESTION

or Copy and paste questions

Prev Next

Powered by
SurveyMonkey
See how easy it is to create a survey.

New version available!

Saving changes...

P21: Interviewe...

Page Logic

More Actions

Cornell University
College of Veterinary Medicine

Copy of Canine Flyball Injury and Nutrition

Interviewer - canine body condition score

120. Please have the interviewer grade your dog's Canine Body Condition Score on 1-9 scale (1= emaciated, 4-5=ideal, 9=extremely obese)

NEW QUESTION

or Copy and paste questions

Prev Next

Powered by

SurveyMonkey
See how easy it is to create a survey.

NEW PAGE

P22: Owner - c...

New version available!

Saving changes...

Cornell University
College of Veterinary Medicine

Copy of Canine Flyball Injury and Nutrition

Owner - canine body condition score

121. Please grade your dog's Canine Body Condition Score on 1-9 scale (1= emaciated, 4-5=ideal, 9=extremely obese)

NEW QUESTION

or Copy and paste questions

Prev Done

Powered by
SurveyMonkey
See how easy it is to create a survey.

NEW PAGE

ENGLISH

About SurveyMonkey • Careers • Developers • Privacy Policy • Email Opt-In •
Copyright © 1999-2019 SurveyMonkey

McAfee SECURE

New version available!

Saving changes...
